# Study on the CID Fragmentation Pathways of Deprotonated 4’-Monophosphoryl Lipid A

**DOI:** 10.3390/molecules26195961

**Published:** 2021-10-01

**Authors:** Ibrahim Aissa, Anikó Kilár, Ágnes Dörnyei

**Affiliations:** 1 Department of Analytical and Environmental Chemistry, Faculty of Sciences, University of Pécs, Ifjúság útja 6, H-7624 Pécs, Hungary; aissa@gamma.ttk.pte.hu; 2 Institute of Bioanalysis, Medical School and Szentágothai Research Centre, University of Pécs, Szigeti út 12, H-7624 Pécs, Hungary; aniko.kilar@aok.pte.hu

**Keywords:** fragmentation pathways, synthetic lipid A, electrospray ionization tandem mass spectrometry

## Abstract

Lipid A, the membrane-bound phosphoglycolipid component of bacteria, is held responsible for the clinical syndrome of gram-negative sepsis. In this study, the fragmentation behavior of a set of synthetic lipid A derivatives was studied by electrospray ionization multistage mass spectrometry (ESI-MS^n^), in conjunction with tandem mass spectrometry (MS/MS), using low-energy collision-induced dissociation (CID). Genealogical insight about the fragmentation pathways of the deprotonated 4’-monophosphoryl lipid A structural analogs led to proposals of a number of alternative dissociation routes that have not been reported previously. Each of the fragment ions was interpreted using various possible mechanisms, consistent with the principles of reactions described in organic chemistry. Specifically, the hypothesized mechanisms are: (i) cleavage of the C-3 primary fatty acid leaves behind an epoxide group attached to the reducing sugar; (ii) cleavage of the C-3’ primary fatty acid (as an acid) generates a cyclic phosphate connected to the nonreducing sugar; (iii) cleavage of the C-2’ secondary fatty acid occurs both in acid and ketene forms; iv) the C-2 and C-2’ primary fatty acids are eliminated as an amide and ketene, respectively; (v) the ^0,2^A_2_ cross-ring fragment contains a four-membered ring (oxetanose); (vi) the ^0,4^A_2_ ion is consecutively formed from the ^0,2^A_2_ ion by retro-aldol, retro-cycloaddition, and transesterification; and (vii) formations of H_2_PO_4_^−^ and PO_3_^−^ are associated with the formation of sugar epoxide. An understanding of the relation between ^0,2^A_2_ and ^0,4^A_2_-type sugar fragments and the different cleavage mechanisms of the two ester-linked primary fatty acids is invaluable for distinguishing lipid A isomers with different locations of a single ester-linked fatty acid (i.e., at C-3 or C-3’). Thus, in addition to a better comprehension of lipid A fragmentation processes in mass spectrometers, our observations can be applied for a more precise elucidation of naturally occurring lipid A structures.

## 1. Introduction

Lipid A is the active component of lipopolysaccharides (also called endotoxins) found in the outer membrane of most gram-negative bacteria [1]. On the one hand, lipid A has been known to affect the immune system, as it is an immunostimulant capable of inducing a protective, non-specific immune response in the host. On the other hand, depending on the dose and the structure of lipid A, the resulting immune response can lead to the uncontrolled production of inflammatory cytokines that may evoke fatal effects, such as high fever, septic shock, and multiorgan failure [2]. In this context, it is not surprising that several lipid A derivatives (synthetic or produced from bacteria) have been targets for developing effective adjuvants in vaccines and immunotherapy formulations [3,4].

Generally, bacterial lipid A comprises of a mixture of molecules, consisting of a β(1→6)-linked diglucosamine backbone, which is variably acylated with acyl or acyloxyacyl groups at the C-2, C-3, C-2’, and C-3’ positions, as well as phosphorylated at the C-1 and/or C-4’ positions [5]. The bisphosphoryl hexa-acyl species (carrying six fatty acyl chains in an asymmetric distribution (4 + 2) over the two glucosamine units), produced by *E. coli*, represents the highest endotoxic activity [6]. Alterations in the acylation and/or phosphorylation pattern, as well as post-translational modifications, significantly influence the immune system recognition (or evasion) [7] and potency of response [8] and may even modulate bacterial resistance to antibiotics [9].

In biological model systems in sepsis research, well-defined reference lipid A products are required to evaluate the cellular response after stimulation with lipid A. The most known synthetic lipid A standard is monophosphoryl lipid A (MPLA), which lacks the phosphate at the C-1 position and the acyl chain at the C-3 position, making it less toxic but with the retained activity of stimulating the adaptive immune response. This ligand is used as an adjuvant and it has been incorporated into several vaccines [10,11]. Besides, other synthetic MPLA structural analogs (such as those used in this study) containing a single molecular species are likely candidates in different adjuvant formulation development studies [4].

In order to accurately decipher the relationship between the functional groups and bioactivity of lipid A, a detailed characterization of its chemical structure is essential. Currently, tandem or multistage mass spectrometry (MS/MS, MS^n^) combined with soft ionization is the most effective method for enhancing structural information [12]. Complete structures of lipid A from a wide range of bacterial strains have been deducted through mass spectrometry alone (i.e., without prior separation or purification of the intrinsically heterogeneous lipid A samples) with the use of different mass analyzers [13,14,15,16,17,18,19,20,21]. Fragmentation rules, described for both negatively and positively charged lipid A molecules, greatly assist in the interpretation of the observed fragment ions [22,23,24,25]. Moreover, energy-resolved mass spectrometry (ERMS) may provide an opportunity to differentiate isobaric lipid A species [26].

Despite the fragmentation behavior of lipid A is reproducible and predictable, the general descriptions of the underlying mechanisms and dependence of fragmentation on specific linkages are rarely described or addressed. Mechanistic investigation, regarding the fragment ion formation by MS^n^, is essential to a better understanding of the ion chemistry and to facilitate the identification of specific product ions, such as cross-ring fragments or those formed by fatty acid eliminations. So far, there are only a few studies in the literature that contain information about the dissociation processes of 4’-monophosphoryl lipid A under low-energy CID conditions. Some of those studies deal with the elimination of the acyl groups closely situated to the phosphate anion [14,27] or chain cleavages taking place at a distance from the localized negative charge [16]. Furthermore, the mechanisms of cross-ring fragment formations for lipid A are scarcely detailed in the literature.

This study aimed to determine the multitude of ions formed during the fragmentation of four synthetic lipid A compounds with structural similarities using an ion trap and a triple quadrupole (QqQ) mass spectrometer with electrospray ionization (ESI), to discover more details about the specific dissociation events and fragmentation routes in the negative-ion mode. The explanation of characteristic ions in MS^n^ mass spectra, obtained by low-energy collision-induced dissociation (CID), could help analysts better employ mass spectral observations for the structural elucidation of unknown lipid A.

## 2. Results and Discussion

### 2.1. MS^2^ Mass Spectra of the [M − H]^−^ Lipid A Precursor Ions

For each synthetic standard, one major peak was detected in the mass spectrum, corresponding to the deprotonated 4’-monophosphoryl lipid A molecule [M − H]^−^ (results not shown). Fragmentation patterns of each standard were studied at the MS^2^ stage, and results obtained with the two mass spectrometers at different RF amplitudes (ion trap) or collision energies (QqQ), respectively, were compared. As can be seen in the case of the deprotonated 3D-PHAD molecule (Figure S1 and S2 in Supplementary Information), there was a difference in the number, appearance, and intensity of fragment ions in the MS^2^ mass spectra from ion trap or QqQ. With the ion trap, mainly the first generation of product ions were produced from the selected precursor ion (*m*/*z* 1518); whereas, with the QqQ (especially when high collision energies were used), several other, higher generation product ions (resulting from a series of competing and consecutive reactions) were also formed. This also meant that sequential MS^n^ analysis of ions, performed with the ion trap, was particularly useful for defining genealogical relationships; however, with the QqQ instrument, the genealogy between ions could not be established. On the other hand, with the QqQ instrument, fragment ions of *m*/*z* below 100 could be scanned; whereas, with the ion trap analyzer (which had a low-mass cut-off value), fragment ions were detected only from *c.a.* 27% of the precursor ion mass. So, the PO_3_^−^ (*m*/*z* 79) and H_2_PO_4_^−^ (*m*/*z* 97) fragment ions could not be observed during the sequential MS^n^ analyses, even though they were formed.

**Figure 1:**
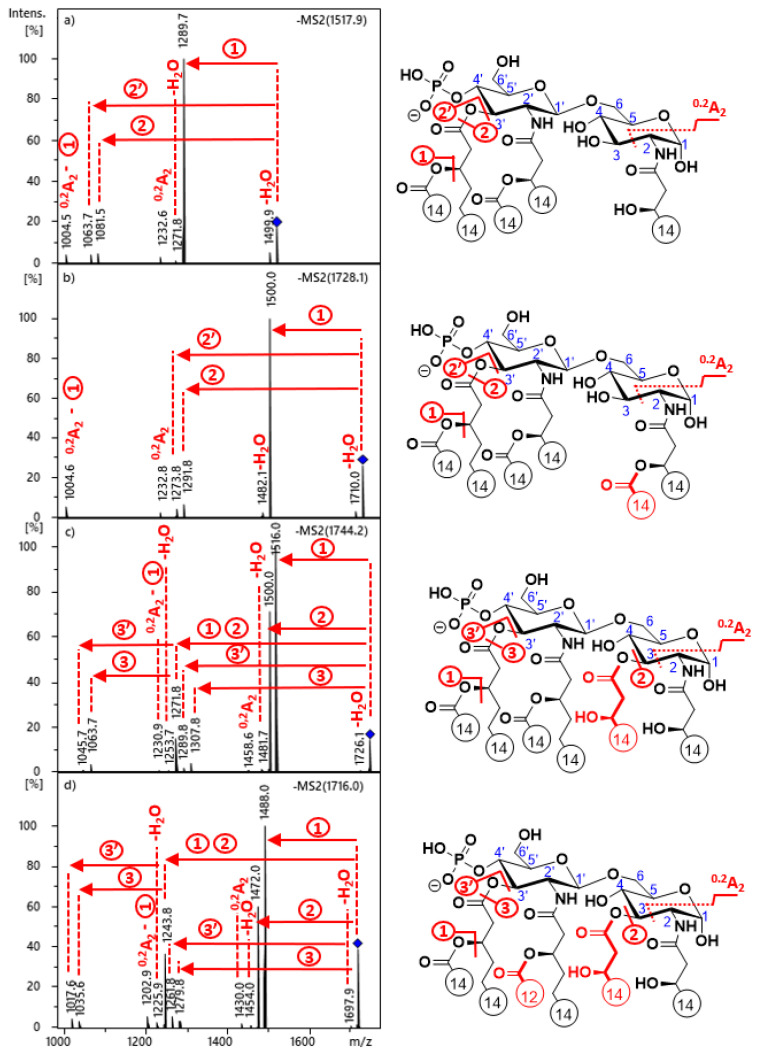
ESI-ion trap MS^2^ mass spectra of the deprotonated 4’- monophosphoryl lipid A molecules of (**a**) 3D-PHAD, (**b**) 3D(6-acyl)-PHAD, (**c**) PHAD, and (**d**) PHAD-504, with the indication of cleavage sites in the structure. Fatty acyl chain lengths are given by numbers. The colored structural parts indicate the differences between the four lipid A congeners.

MS^2^ mass spectra from ion trap of the [M − H]^−^ precursor ions of the four lipid A standards are shown in Figure 1. The identity of fragment ions was determined by applying fragmentation rules (described previously) for the CID dissociation of lipid A molecules in the deprotonated form [23]. For each standard, the most intense peak (i.e., appearing at *m*/*z* 1290 for 3D-PHAD, *m*/*z* 1500 for 3D-(6-acyl)-PHAD, *m*/*z* 1516 for PHAD, and *m*/*z* 1488 for PHAD-504) was generated by releasing the fatty acid (C14:0, 228 u) from the C-3’ secondary position, known as the most labile cleavage site of 4’-monophosphoryl lipid A at low collision energies [23]. Next to this peak, another intense peak appeared for the 3-acyl standards (at *m*/*z* 1500 and *m*/*z* 1472 in Figure 1c,d, respectively), due to the elimination of a C14:0(3-OH) (Δ*m* = 244 u) fatty acid from the C-3 primary position, known as the second most labile cleavage site [23]. Apart from these two, other types of single fatty acid loss from the precursor were not observed for the synthetic lipid A congeners used in this study. Meanwhile, in previous studies on bacterial-derived 4’-monophosphoryl lipid A species (such as from *E. coli*), usually, a third fragment was also detected arising from the fatty acid loss at the C-2’ secondary position from the precursor. If this were the case for the PHAD-504 standard, which has the same structure as the 4’-monophosphoryl lipid A species produced by *E. coli* (and the only structure among the standards in which the type of oxyacyl groups linked at C-2’ and C-3’were different), a fragment ion peak would be expected at *m*/*z* 1516. However, the lack of any signal at *m*/*z* 1516 in Figure 1d was a straightforward indication of the lack of release of the lauric acid (C12:0, 200 u) from the C-2’ branched position from the pure, synthetic sample. A conceivable explanation of the peak at *m*/*z* 1516, detected by others for various *E. coli*-type lipid A species [14,16], is that a structural isomer, i.e., the 1-monophosphoryl species may also have been present in the native samples. From that isomer, the fatty acid loss from the C-2’ secondary position is the most preferred cleavage process, based on previous observations on the CID fragmentation of lipid A phosphorylation isomers [24]. Furthermore, it should be noted that the complete lack of release of the C-2’ branched-chain in 4’-monophosphoryl lipid A may also have importance in the unambiguous identification of first-generation product ions from lipid A species having the same type of C-2’ and C-3’ secondary fatty acids, thus eliminating the theoretical possibility of multiple fragmentation routes, leading to the suggestion of various diastereomeric structures.

For the 3-acyl standards, another peak with moderate intensity appeared (at *m*/*z* 1272 and 1244 in Figure 1c,d, respectively), which derived from the combined losses of the C-3’ secondary and C-3 primary fatty acids (Δ*m* = 472 u). From this latter fragment, further loss of a C14:1, as a ketene or an acid (Δ*m* = 208 or 226 u, respectively) from the C-3’ primary position, resulted in pairs of low-intensity fragment ions (at *m*/*z* 1064 and *m*/*z* 1046 in Figure 1c and *m*/*z* 1036 and *m*/*z* 1018 in Figure 1d).

The loss of the C-3’ linked C14:0(3-O-C14:0) acyloxyacyl chain, both as a ketene or an acid (Δ*m* = 436 or 454 u), gave rise to peak pairs, detected with very low intensities for all precursor ions (at *m*/*z* 1082/1064, *m*/*z* 1292/1274, *m*/*z* 1308/1290, and *m*/*z* 1280/1262 in Figure 1a–d, respectively). Water losses (Δ*m* = 18 u) were detected with low intensities, both from the precursors and some acyl loss fragment ions. It should also be noted that neither the release of the C-2 secondary fatty acid nor releases of the amide-linked primary fatty acids were observed. Although, these were not surprising because losses of those substituents are usually less preferential at the MS^2^ stage [18,23].

In each MS^2^ mass spectrum, several ^0,2^A_2_ diagnostic cross-ring fragments appeared, although with relatively low intensities. As the nonreducing GlcN structures of the 3-deacyl standards were comparable, they presented the same ^0,2^A_2_ fragment ion peak pairs (at *m*/*z* 1233 and *m*/*z* 1005 in both Figure 1a,b), of which the latter was formed after the loss of a C14:0 from the C-3’ secondary position. On the other hand, different ^0,2^A_2_-type ions were detected for the 3-acyl standards (at *m*/*z* 1459 and *m*/*z* 1231 in Figure 1c and *m*/*z* 1431 and *m*/*z* 1203 in Figure 1d), preceding and proceeding the C-3’ secondary acyl loss, respectively.

Next, all the above-mentioned fragment ions were subjected to sequential mass analysis measurements in the ion trap instrument. The MS^n^ mass spectra (n = 3–6) revealed further cleavage products, such as ^0,4^A_2_-type cross-ring fragments and those formed by the release of fatty acids from the C-2’ secondary and C-2’ and C-2 primary positions. In the following chapters, a selection of fragment ions will be used to elucidate details on the prominent fragmentation pathways and propose the mechanistic origin of the lipid A product ions detected in the negative-ion mode.

### 2.2. Proposed Mechanisms for the Release of the Secondary Fatty Acids

**Scheme 1:**
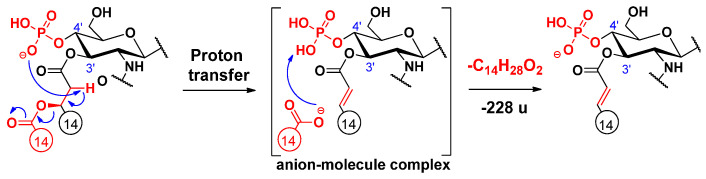
Proposed fragmentation mechanism for the loss of the C-3’ branched acyl chain [16].

An alternative elimination mechanism of the C-3’ oxyacyl group (as free fatty acid), adjacent to the phosphate group, has already been explained by Madalinski et al. [16] using a charge-driven dissociation via the formation of an anion–molecule complex (Scheme 1). Namely, the phosphate group (basic site) removes a proton from the α-position (acidic site) of the primary fatty acyl chain linked at the C-3’ position, then an α–β elimination is followed by the formation of an intermediate anion–molecule complex.

**Scheme 2:**
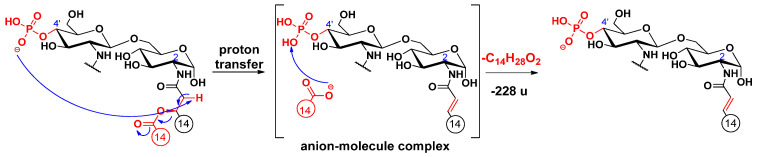
Proposed fragmentation mechanism for the loss of the C-2 branched acyl chain (as an acid).

**Scheme 3:**
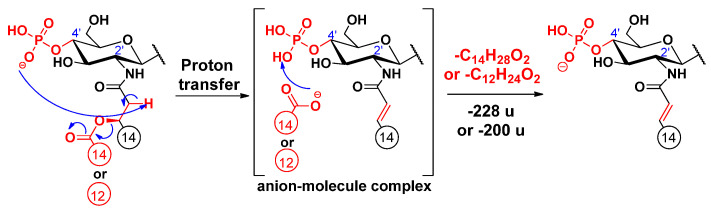
Proposed fragmentation mechanism for the loss of the C-2’ branched acyl chain (as an acid).

**Scheme 4:**
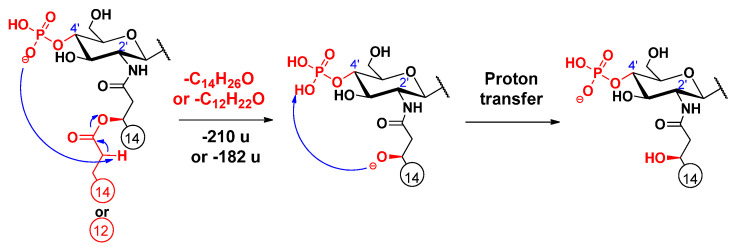
Proposed fragmentation mechanism for the loss of the C-2’ branched acyl chain (as a ketene).

Cleavages of the secondary fatty acids at C-2’ or C-2 were only observed in higher MS^n^ stages (n = 4, 5, 6), after most of the labile ester-linked fatty acids had been released and/or cross-ring fragmentation(s) had occurred (for representative examples, see Figure S3 and S4 in Supplementary information). Similar to the previously described cleavage mechanism of the C-3’ oxyacyl group (vide supra), we propose similar processes for the eliminations of the C-2 and C-2’ secondary fatty acids (Scheme 2 and Scheme 3, respectively), proceeding with the release of the C-3’ primary fatty acid as a ketene. Only the proton transfer step is different, as the acidic site is at the α-position of the respective amide group, and the basic site is the phosphate ion at C-4’. Furthermore, according to the MS^n^ measurements, the C-2’ secondary fatty acid was also eliminated in ketene form (Figure S5). The elimination can be explained with an intramolecular hydrogen transfer from the α-carbon of the ester group and the direct O–CO bond cleavage occurring without anion–molecule complex formation (Scheme 4) (for eliminations proceeding the release of the C-3’ primary fatty acid as an acid, see Schemes S1–S3). Apart from the product ions assigned to the secondary fatty acyl eliminations, the released secondary fatty acids could also be detected in deprotonated forms (e.g., *m*/z 227 for a C14:0 in Figure S2) but only with very low intensities and under relatively high-energy collisional conditions.

Furthermore, it should be noted that the precursor ions (shown in Scheme 2, Scheme 3 and Scheme 4) may have even different structures. Either a double bond is present in the sugar ring (due to loss of the C-3’ linkage) and the negative charge is on the P–O oxygen atom or it involves a cyclic phosphate species carrying the negative charge, as suggested for the first time in this study (vide infra).

### 2.3. Proposed Mechanisms for the Release of the Primary Fatty Acids

**Scheme 5:**
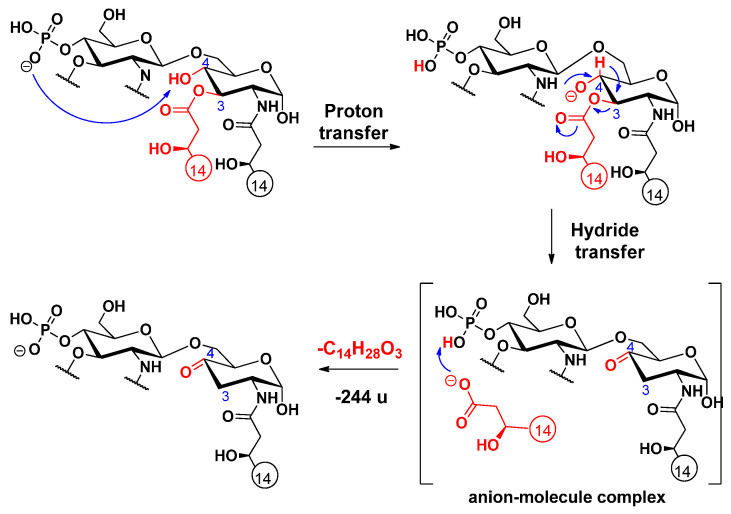
Proposed fragmentation mechanism for the loss of the C-3 primary fatty acid, leading to the formation of a ketone group in the sugar ring [16].

**Scheme 6:**
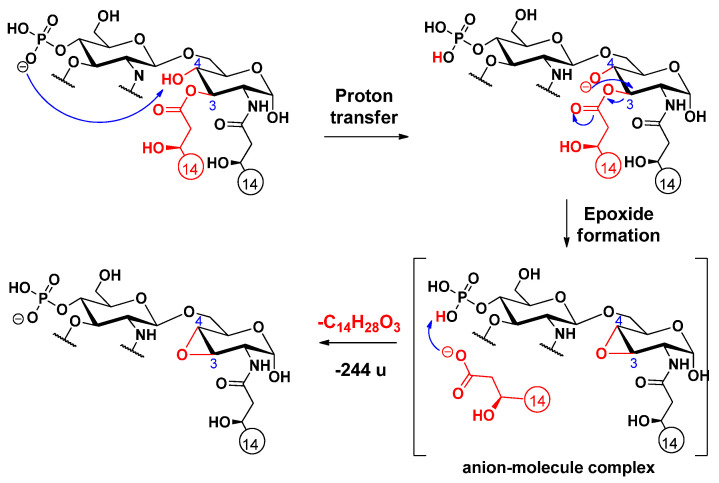
Proposed fragmentation mechanism for the loss of the C-3 primary fatty acid, leading to the formation of a sugar epoxide.

Cleavage of the C-3 linked primary fatty acyl chain was observed for the 3-acyl lipid A molecules: PHAD and PHAD-504. Unlike the C-3’ linked primary fatty acid, which can leave both as an acid and a ketene (vide infra), the C-3 linked fatty acyl chain is lost only as an acid. The first step of this charge-driven process (induced by a long proton transfer, as proposed before [16]) is the formation of a reactive alkoxide group at the C-4 position. Next, either a ketone derivative can be formed (Scheme 5), as previously reported [16], or an epoxide derivative may develop (Scheme 6), as proposed here (it should be noted that epoxide sugars are versatile intermediates in organic synthesis [28]). While the former involves an anion–molecule complex formation by hydride transfer, the latter product ion assumes an anion–molecule complex, resulting from the anti-attack of the alkoxide ion at C-4 on the hydroxymyristic acid chain at C-3.

The primary fatty acid was eliminated from the C-3’ position as an unsaturated primary fatty acid (C14:1) in all standards (observe that the preceding elimination of the C-3’ branched-chain leaves behind a double bond). Regularly, the loss of this primary substituent occurs in two forms, as a ketene and an acid, by two competing processes, resulting in diagnostic peak pairs with 18 u apart in the mass spectrum.

**Scheme 7:**
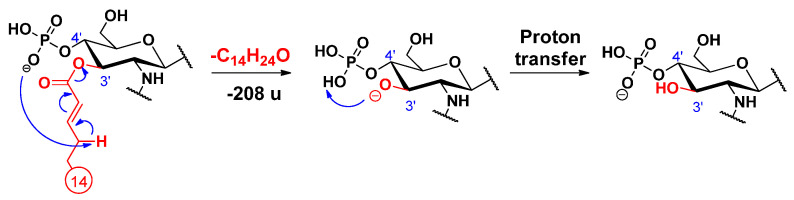
Proposed fragmentation mechanism for the loss of the C-3’ primary fatty acid residue as a ketene, along with a proton transfer.

For the ketene loss, a charge-driven fragmentation process has been suggested earlier [16]. In our case, this type of process is initiated with a proton removal (where the proton is uptaken by the anionic charge of the phosphate group) from the allylic position of the unsaturated chain; then, the conjugated carbanion promotes the direct O–CO bond cleavage, leading to the release of the unsaturated alkyl chain as ketene (Scheme 7). In addition, we hypothesize that a proton transfer takes place, and the negative charge is held by the phosphate group in the end.

**Scheme 8:**
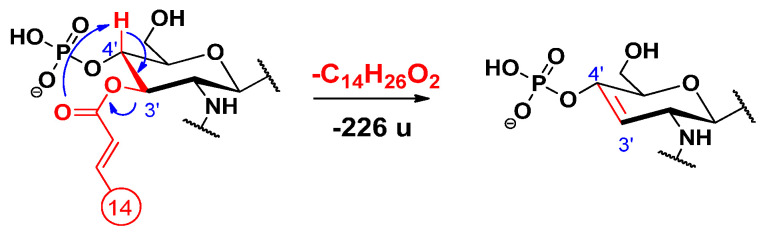
Proposed fragmentation mechanism for the loss of the C-3’ primary fatty acid residue as an acid (as described before [14]).

**Scheme 9:**
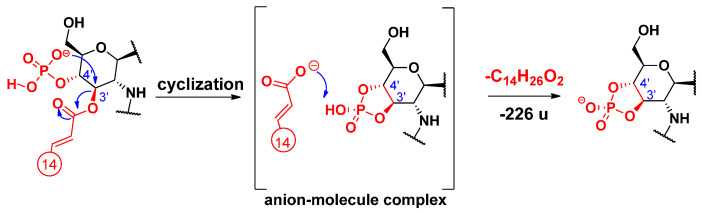
Proposed alternative fragmentation mechanism for the loss of the C-3’ primary fatty acid residue as an alkyl carboxylic acid.

For loss of the C-3’ primary chain as a free fatty acid, a charge-remote fragmentation process has been proposed [14], in which the removal of a hydrogen atom at C-4’ led to the formation of a double bond between the C-3’ and C-4’ carbon atoms (Scheme 8). Besides, we propose here an alternative, charge-driven dissociation pathway. Namely, a five-membered cyclic phosphate is generated in a process initiated by the attack of the anionic charge site of the phosphate group on the carbon C-3’, leading to the removal of the unsaturated alkyl chain as a free fatty acid (Scheme 9). In fact, this process is very similar to what was observed for cyclic phosphate anions of diacyl glycerophosphoethanolamines [29].

**Scheme 10:**
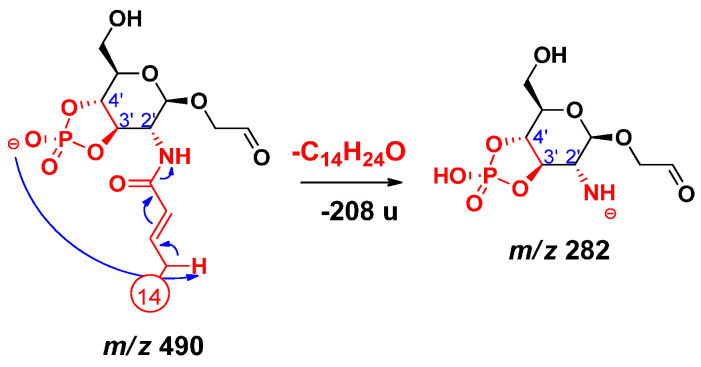
Proposed fragmentation mechanism leading to the release of the residual C-2’ primary fatty acid as ketene.

Elimination of the amide-linked primary fatty acid at C-2’ was observed only in the case of 3D-PHAD, having the lowest molecular weight among the four standards. Here, a fragment ion at *m*/*z* 282 (Figure S5) was formed by eliminating a residual C14:1 from the C-2’ position as ketene (Δ*m* = 208 u). The proposed mechanism starts with a proton transfer from the allylic position of the amide-linked unsaturated alkyl chain to the phosphate group, either to the cyclic one (as shown in Scheme 10) or to the noncyclic alternative (Scheme S4). Then, the conjugated carbanion promotes the direct N–CO bond cleavage.

**Scheme 11:**
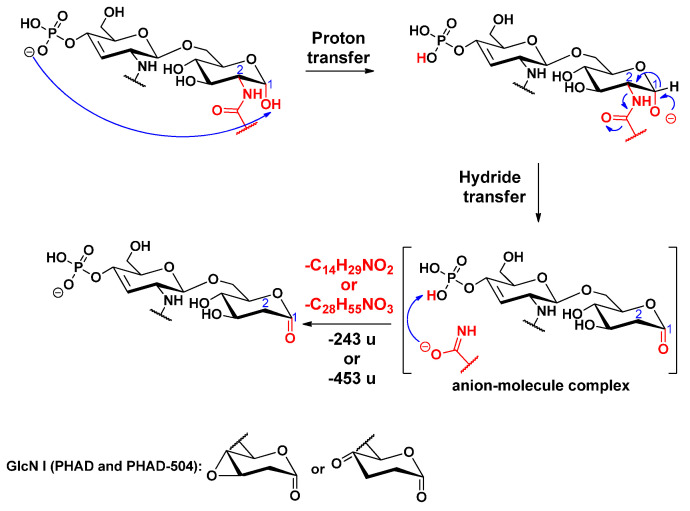
Proposed fragmentation mechanism for the loss of the C-2 primary fatty acid, leading to the formation of a ketone group in the sugar ring.

Cleavage of the amide-linked C-2 primary fatty acyl chain was observed for all standards. An example is shown for PHAD-504 (Figure S4), where the fragment at *m*/*z* 774 was formed by elimination of the hydroxymyristic acid amide (Δ*m* = 243 u) from the ion at *m*/*z* 1017. The first step of this charge-driven process (induced by a long proton transfer) is the formation of a reactive alkoxide group at the C-1 position. Next, a ketone derivative is formed by a hydride transfer via the formation of an anion–molecule complex (Scheme 11).

### 2.4. Cross-ring Fragmentations

**Figure 2:**
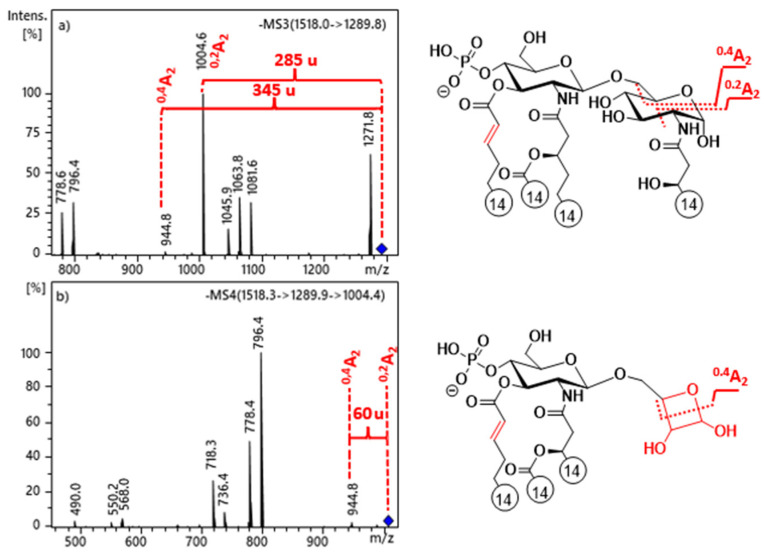
ESI-ion trap mass spectra obtained at MS^3^ and MS^4^ stages of selected ions at (**a**) *m*/*z* 1290 and (**b**) *m*/*z* 1005 for 3D-PHAD, demonstrating the structural relationship between ^0,2^A_2_ and ^0,4^A_2_ cross-ring fragment ions in 3-deacyl lipid A.

In the MS^3^ spectra of selected precursors, *m*/*z* 1290 of 3D-PHAD (Figure 2a) and *m*/*z* 1500 of 3D(6-acyl)-PHAD (Figure S6a), both of which lack the C-3’ secondary acyl chain, two types of cross-ring fragment ions were detected: ^0,2^A_2_ (e.g., at *m*/*z* 1005) and ^0,4^A_2_ (at *m*/*z* 945). However, the ^0,4^A_2_ fragment ion was also present in the MS^4^ spectrum of the ^0,2^A_2_ ion selected as a precursor (see Figure 2b for 3D-PHAD and Figure S6b for 3D(6-acyl)-PHAD). This observation suggests that a structural relationship exists between the two cross-ring fragments. As the mechanisms of cross-ring fragment formations for lipid A are not very well detailed in the literature, we took these dissociations under investigation.

**Scheme 12:**
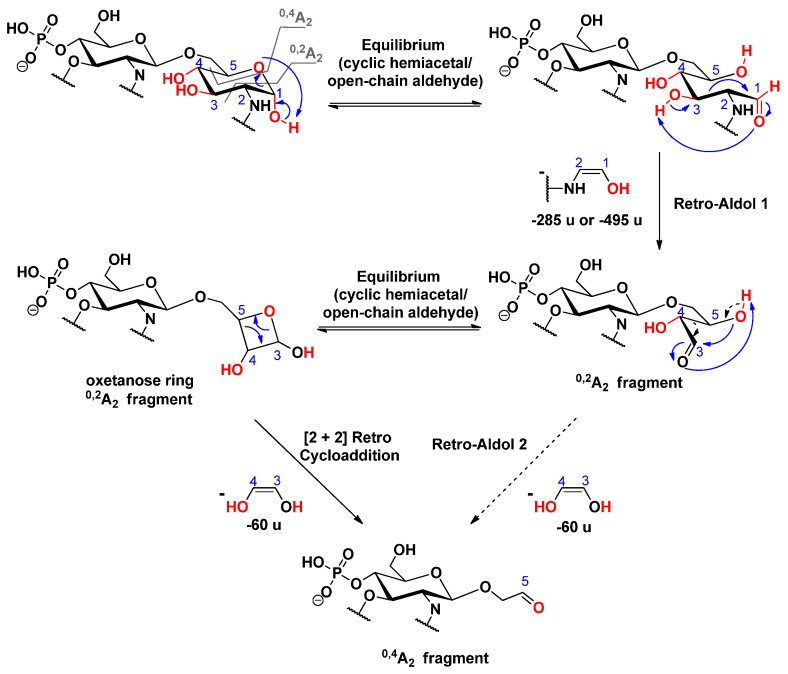
Proposed mechanisms for the consecutive formation of ^0,2^A_2_ and ^0,4^A_2_ fragment ions in 3-deacyl lipid A.

Generally, ^0,2^A_2_ and ^0,4^A_2_ cross-ring fragments are simultaneously detected in the MS^2^ mass spectra of 4’-monophosphoryl lipid A species, in which the C-1 position is free [25]. Thus, it can be assumed that the first step in the mechanism of A-type cross-ring cleavages involves ring-opening [30] of the reducing sugar, in order to produce an equilibrium amount of the open-chain aldehyde, which is then capable of acting as a reducing agent. Consequently, this form can undergo a retro-aldol reaction [31], in order to yield a shorter chain of α,β-dihydroxy aldehyde (i.e., ^0,2^A_2_ fragment) via the neutral loss of the anchored enol (Scheme 12). For the further decomposition of the ^0,2^A_2_ product ion, we suggested two pathways: (i) either a second retro-aldol reaction takes place and leads directly to the ^0,4^A_2_ fragment ion by loss of an ethane-1,2-diol (Δ*m* = 60 u); or (ii) the hydroxyl group attached to C-5 reacts with the formed aldehyde, which, by a second hemiacetal-aldehyde equilibrium, forms a four-membered ring, so-called oxetanose [32], which can undergo a [2 + 2] retrocycloaddition reaction [33] (Scheme 12). The predominance of the oxetanose derivative is also supported by the study of Lee et al. [34], in which they showed via synthesis of diacylglycerol analogs that a four-membered ring (oxetanose) was obtained in equilibrium, with its corresponding open-chain form (nonoxetanose) at a ratio of 5:1.

**Figure 3:**
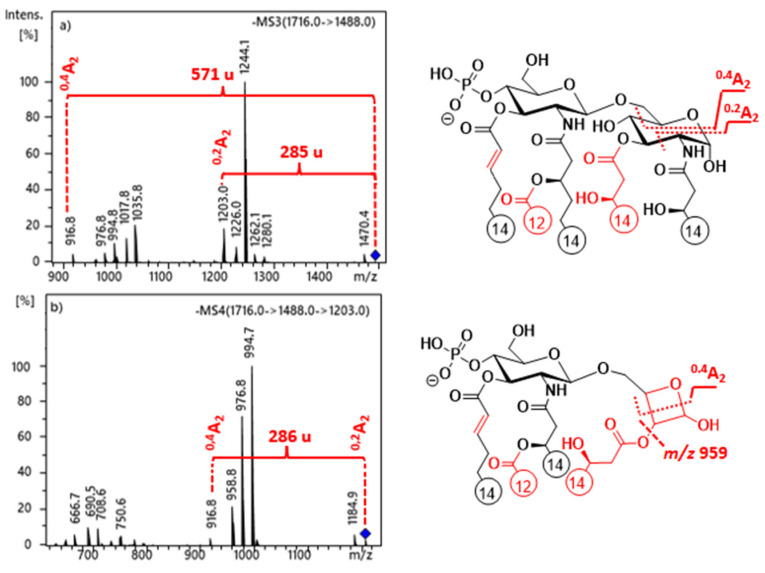
ESI-ion trap mass spectra obtained at MS^3^ and MS^4^ stages of selected ions at (**a**) *m*/*z* 1488 and (**b**) *m*/*z* 1203 for PHAD-504, demonstrating the structural relationship between ^0,2^A_2_ and ^0,4^A_2_ cross-ring fragment ions in 3-acyl lipid A.

**Scheme 13:**
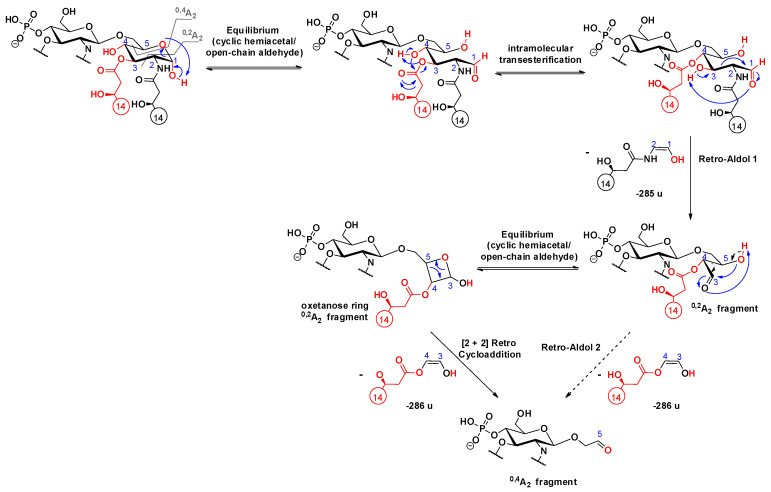
Proposed mechanisms for the consecutive formation of ^0,2^A_2_ and ^0,4^A_2_ fragment ions in 3-acyl lipid A.

In the MS^3^ spectra of selected precursors, *m*/*z* 1488 of PHAD-504 (Figure 3a) and *m*/*z* 1516 of PHAD (Figure S7a), both of which had lost the C-3’ secondary acyl chain, signals originating from ^0,2^A_2_ and ^0,4^A_2_ cross-ring fragmentations (*m*/*z* 1203 and 917 for PHAD-504, and *m*/*z* 1231 and 944 for PHAD, respectively) appeared, as well. Furthermore, a relationship between the ^0,2^A_2_ and ^0,4^A_2_ ions was observed in the MS^4^ spectrum of the chosen ^0,2^A_2_ precursor ion (Figure 3b for PHAD-504, Figure S7b for PHAD). Due to the presence of an additional C14:0(3-OH) chain at C-3 in the reducing sugar of these molecules, the proposed mechanism of cross-ring cleavages is one step different from that described above for the 3-deacyl lipid A derivatives. Namely, following the first step (i.e., ring-opening of the reducing sugar), an intramolecular transesterification occurs between the hydroxyl group at C-4 and the ester chain at C-3, leading to a derivative that can undergo a retro-aldol reaction. Thus, it is suggested that the ^0,4^A_2_ fragment be formed, either from a second retro-aldol or a [2 + 2] retro-cycloaddition reaction (Scheme 13).

In addition, a small signal appeared at *m*/*z* 959 in Figure 3b, resulting from the release of the fatty acyl chain from the oxetanose ring in the ^0,2^A_2_ fragment. Indeed, the cleavage of this fatty acyl chain (originally linked at C-3 in lipid A) may occur from the ^0,2^A_2_ fragment, but only with a small probability. Additionally, note that when the C-3 primary fatty acid had been eliminated (i.e., preceding the cross-ring fragmentation), the ^0,2^A_2_ fragment cannot be formed at all [14].

In summary, the observation that there is a structural relationship between the two A-type cross-ring fragments and those intensity ratios between them are different in the case of 3-acyl and 3-deacyl lipid A also implies that one may easily distinguish the simultaneous presence of C-3 or C-3’ acylation isomers in a single sample. Namely, the appearance of low-intensity ^0,2^A_2_ fragments in the mass spectrum is characteristic of a 3-acyl, 3’-deacyl lipid A; meanwhile, high-intensity ^0,2^A_2_ fragments appearing as peak pairs (with 18 u apart) are typical for a 3-deacyl, 3’-acyl lipid A. Without applying these “rules”, misleading information of some product ions in complex lipid A mixtures may be obtained, and some low abundance, but biologically important, acylation isomers can remain unexplored [23,35].

### 2.5. Dephosphorylation

Two signals, at *m*/*z* 79 and 97, corresponding to the monometaphosphate (PO_3_^−^) and dihydrogen phosphate (H_2_PO_4_^−^) ions, respectively, were observed in the MS^2^ mass spectra from QqQ of the four lipid A precursor ions (an example for 3D-PHAD is shown in Figure S2). As both fragment ions were related to the C-4’ phosphate moiety, we took mechanisms leading to their formation under investigation. In the literature, the mechanism of dephosphorylation reaction of lipid A under acidic conditions has only been proposed for the anomeric phosphate group (at position C-1) [16].

**Scheme 14:**
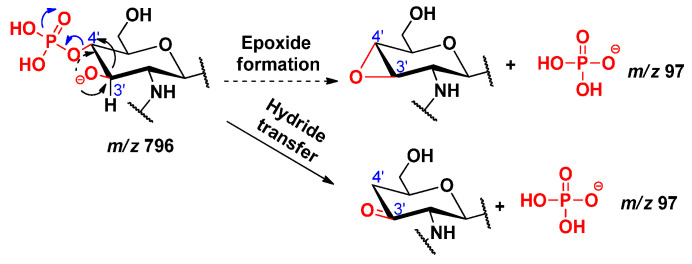
Proposed mechanisms for the formation of dihydrogen phosphate ion H_2_PO_4_^−^.

**Scheme 15:**
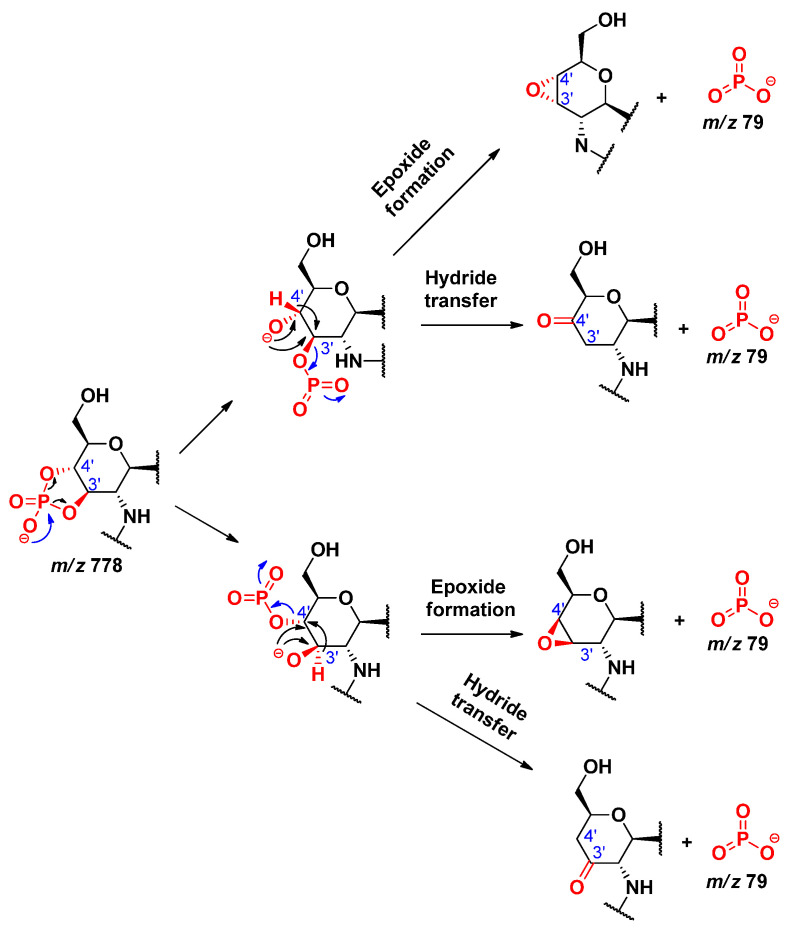
Proposed alternative routes for the formation of metaphosphate ion, PO_3_^−^, from the cyclic phosphate lipid A derivative.

**Scheme 16:**
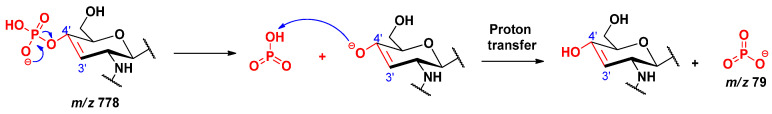
An unlikely mechanism for the formation of metaphosphate ion, PO_3_^−^, from the noncyclic phosphate lipid A derivative.

By comparing the MS^2^ mass spectra related to different collision energies in Figure S2, it could be seen that as the collision energy increased, the relative intensity of the peaks at *m*/*z* 796 and 778 decreased, while that of the peaks at *m*/*z* 79 and 97 increased. The peaks at *m*/*z* 796 and 778 each correspond to an ^0,2^A_2_ fragment, which had lost the C-3’ acyloxyacyl group either as a ketene or an acid, respectively. The formation of dihydrogen phosphate ion (H_2_PO_4_^−^, *m*/*z* 97) was considered from the fragment at *m*/*z* 796, by two possible pathways (Scheme 14). In the first way, an epoxidation takes place (involving the antiperiplanar relationships between the formed oxide ion at C-3’ and the phosphate group), which will then easily promote the removal of the H_2_PO_4_ that will hold the negative charge. In the second way, a hydride transfer takes place, leading to the formation of the H_2_PO_4_^−^ ion. The formation of monometaphosphate ion (PO_3_^−^, *m*/*z* 79) was considered from the fragment at *m*/*z* 778, by two pathways, starting from the cyclic phosphate derivative (Scheme 15), from which the monometaphosphate ion could be considered as a good leaving group, either following a hydride transfer or an epoxide formation. However, the pathway starting from the non-cyclic phosphate derivative (Scheme 16) was not considered, as in that case, the PO_3_^−^ would be formed via the removal of a proton from the detached neutral metaphosphoric acid (HPO_3_), which would lead to detection of the intermediate fragmentation product, i.e., the negatively charged lipid A, without the C-4’ phosphate group; albeit, such a phosphate-free product ion (*m*/*z* 698) was not seen in the MS^n^ mass spectra.

## 3. Experimental

### 3.1. Chemicals

Methanol and dichloromethane (LC-MS Chromasolv grade) were purchased from Sigma–Aldrich (Steinheim, Germany).

### 3.2. Lipid A Samples

Four types of synthetic monophosphoryl lipid A standards were purchased from Sigma–Aldrich, the exclusive Avanti Polar Lipids provider (Alabaster, AL, USA) in Hungary. The standards were monophosphoryl 3-deacyl penta-acyl lipid A (3D-PHAD, Pat No. 9,241,988), monophosphoryl 3-deacyl hexa-acyl lipid A (3D(6-acyl)-PHAD), monophosphoryl lipid A (PHAD), and monophosphoryl lipid A-504 (PHAD-504). The standards were purchased in their ammonium salt forms.

### 3.3. Lipid A Sample Preparation

A few amounts (0.1 mg in powder form) of each synthetic lipid A were introduced into Eppendorf tubes and suspended in a mixture of 900 μL methanol and 100 μL dichloromethane. The samples were vortexed and subsequently put in an ultrasonic bath (5 min) to be highly homogenous. Then, 100 μL of each sample was introduced into sealed glass vials, followed by the addition of 900 μL of methanol. After vortexing, the samples were ready for injection.

### 3.4. Mass Spectrometric Analysis

ESI-ion trap MS^n^ analysis was performed in the negative-ion mode using an LC/MSD Trap XCT Plus mass spectrometer (Agilent Technologies, Waldbronn, Germany), controlled with the Agilent LC/MSD Trap Soft-ware 5.3. A syringe pump, set at a flow rate of 3 µL/min, was used for direct infusion of the samples into the ion source. The electrospray capillary high voltage was applied at 3500 V. Nitrogen was used both as nebulizer (15 psi, 5 L/min) and dry gas (at 325 °C). The smart parameter setting (SPS) of the software was utilized to simplify the ion-trap operation, i.e., the target mass was set at the mass of the deprotonated lipid A, and both compound stability and trap drive level were set at 100%. Mass spectra were scanned in the range of *m*/*z* 100–2200, with a scan speed of 8100 *m*/*z* per second. The mass isolation window for precursor ion selection was set to 2 *m*/*z* in the multiple-stage MS analyses. Each precursor ion was excited by resonant excitation voltage, fixed between 0.5 V and 1.5 V, according to the intensity of the parent ion signal. Negative-ion mass spectra, including collision-induced dissociation (CID) spectra, were averaged over 5–20 scans, depending on the relative abundances of the precursor ions.

ESI-QqQ measurements were performed in the negative-ion mode using a SCIEX Triple Quad^TM^ 6500+ System (AB Sciex LP, Concord, ON, Canada), equipped with an integrated syringe pump set at a flow rate of 25 µL/min. Mass spectra were scanned in the range of *m*/*z* between 50 and 1600. The scan rate was 1000 for both Q1 MS and product ion MS^2^. The ion spray voltage and temperature were set at –4500 V and 200 °C, respectively. The curtain gas was set at a flow rate of 20 psi, gas 1 (nebulizer gas) was set at 3 psi, and gas 2 (heater gas) was set at 0 psi. The collision gas (CAD) was set at 8. Data were acquired using Analyst software 1.7.2.

### 3.5. Data Evaluation

The evaluation of the MS^n^ mass spectra was made by considering the monoisotopic masses of the fragments ions and the neutral losses, based on the known composition and structure of the precursor ions. The following abbreviations were used to indicate the chemical compositions during the interpretation of mass spectra: GlcN (2-amino-2-deoxy-glucopyranose), C12:0 (dodecanoic acid or lauric acid), C14:0(3-OH) (3-hydroxytetradecanoic acid or hydroxymyristic acid), C14:0 (tetradecanoic acid or myristic acid), and C14:1 (tetradecenoic acid or unsaturated myristic acid). The classical nomenclature for glycoconjugates and cross-ring fragmentation nomenclature, described by Domon and Costello [36], was adopted to designate the fragment ions.

## 4. Conclusions

The objective of the present paper was to propose alternative dissociation mechanisms of deprotonated 4’-monophosphoryl lipid A species, as the generation of particular product ions in gas-phase has been scarcely explained in the literature. Mechanisms proposed here consider both single and multistep cleavages, with rearrangements occurring either by proton or hydride transfer and cyclization. Multistep fragmentation experiments (with ESI-ion trap) were especially useful for ascertaining the relationship between ^0,2^A_2_ and ^0,4^A_2_ cross-ring fragment ions, while ESI-QqQ (with varying energy levels) permitted the finding of a relationship between product ions related to dephosphorylation. By applying low-energy conditions, mainly charge-induced processes were considered for the release of the different ester- or amide-linked fatty acyl chains. Besides providing a better understanding of lipid A fragmentation processes, our observations may also have some potential applications in the interpretation of the mass spectra of naturally occurring lipid A compounds. For instance, the preferential loss of the secondary acyl group at C-3’ over that at C-2’ provides important information on the structure of 4’-monophosphoryl lipid A, carrying the same secondary substituents at these positions. Moreover, as first-generation product ions formed by the release of the C-2’ secondary fatty acyl chain are not favored in 4-monophosphoryl species (unlike 1-monophosphoryl lipid A); thus, the detection of both C-3’ and C-2’ deficient first-generation fragments in the mass spectrum indicates the presence of phosphorylation isomers in the sample. Furthermore, the detection of several pairs of peaks, with 18 u apart in the MS/MS or higher stage MS^n^ mass spectra, in combination with relatively high-intensity ^0,2^A_2_ fragments, indicates the presence of a 3’-acyl, 3-deacyl lipid A compound in the sample.

## Data Availability

Data are contained within the article and Supplementary Materials.

## Supplementary Materials

The following are available online, Figure S1: ESI-ion trap MS2 mass spectra of the deprotonated form of the 3D-PHAD lipid A molecule (precursor ion m/z 1518), applying different RF amplitudes; Figure S2: ESI-QqQ MS2 mass spectra of the deprotonated form of the 3D-PHAD lipid A molecule (precursor ion m/z 1518), applying different collision energies (CE); Figure S3: ESI-ion trap mass spectra, obtained at MS4, MS5, and MS6 stages for several fragment ions of 3D-PHAD, demonstrating the release of myristic acid (Δm = 228u) as an acid from the C-2’ secondary position; Figure S4: ESI-ion trap mass spectra, obtained at MS4 and MS5 stages for several fragment ions of PHAD-504, demonstrating the release of lauric acid (Δm = 200 u) as an acid from the C-2’ secondary position, and the release of hydroxymyristic acid amide from the C-2 primary position (Δm = 243 u); Figure S5: ESI-ion trap mass spectra, obtained at (a) MS4 stage of the selected ion at m/z 718 for 3D-PHAD, demonstrating the release of myristic acid (Δm = 210 u) as a ketene from the C-2’ secondary position, and the release of unsaturated myristic acid (Δm = 208 u) as a ketene from C-2’ primary position; and (b) at MS5 stage of the selected ion at m/z 690 for PHAD-504, demonstrating the release of lauric acid (Δm = 182 u) as a ketene from the C-2’ secondary position; Figure S6: ESI-ion trap mass spectra, obtained at MS3 and MS4 stages of selected precursor ions at (a) m/z 1500 and (b) m/z 1005 for 3D(6-acyl)-PHAD, demonstrating the structural relationship between 0,2A2 and 0,4A2 cross-ring fragment ions in 3-deacyl lipid A; Figure S7: ESI-ion trap mass spectra, obtained at MS3 and MS4 stages of selected precursor ions at (a) m/z 1516 and (b) m/z 1231 for PHAD, demonstrating the structural relationship between 0,2A2 and 0,4A2 cross-ring fragment ions in 3-acyl lipid A; Scheme S1: Alternative fragmentation pathways for the elimination of the C-2 branched acyl chain as an acid in lipid A; Scheme S2: Alternative fragmentation pathways for the elimination of the C-2’ branched acyl chain as an acid in lipid A; Scheme S3: Alternative fragmentation pathway for the C-2’ branched acyl chain as a ketene in lipid A; Scheme S4: Proposed mechanism leading to the release of the C-2’ primary fatty acid as ketene in lipid A.

## Sample Availability

Samples of the compounds are not available from the authors.
