# Supplementary material for: Study on the CID Fragmentation Pathways of Deprotonated 4’-Monophosphoryl Lipid A
Source: Molecules. 2021 Oct 1;26(19):5961. doi: 10.3390/molecules26195961 (PMC8512036; doi:10.3390/molecules26195961)
Supplement: Supplementary file 1 [file molecules-26-05961-s001.zip › molecules-1378580-supplementary.pdf]

Study on the CID fragmentation pathways of deprotonated 4'-monophosphoryl lipid A

Ibrahim Aissa[a], Anikó Kilár[b], Ágnes Dörnyei[a*]

[a] Department of Analytical and Environmental Chemistry, Faculty of Sciences, University of Pécs, Ifjúság útja 6, H-7624 Pécs, Hungary.

[b] Institute of Bioanalysis, Medical School and Szentágothai Research Centre, University of Pécs, Szigeti út 12, H-7624 Pécs, Hungary

Corresponding Author

* E-mail: dornyei@gamma.ttk.pte.hu

Contents

Figure S1. ESI-ion trap MS^2 mass spectra of the deprotonated form of the 3D-PHAD lipid A molecule (precursor ion *m/z* 1518) applying different RF amplitudes.......... 2

Figure S2. ESI-QqQ MS^2 mass spectra of the deprotonated form of the 3D-PHAD lipid A molecule (precursor ion *m/z* 1518) applying different collision energies (CE). 3

Figure S3. ESI-ion trap mass spectra obtained at MS^4, MS^5 and MS^6 stages for several fragment ions of 3D-PHAD, demonstrating the release of myristic acid (Δm = 228u) as an acid from the C-2' secondary position.......... 4

Figure S4. ESI-ion trap mass spectra obtained at MS^4 and MS^5 stages for several fragment ions of PHAD-504, demonstrating the release of lauric acid (Δm = 200 u) as an acid from the C-2' secondary position, and the release of hydroxymyristic acid amide from the C-2 primary position (Δm = 243 u). 5

Figure S5. ESI-ion trap mass spectra obtained at a) MS^4 stage of the selected ion at *m/z* 718 for 3D-PHAD, demonstrating the release of myristic acid (Δm = 210 u) as a ketene from the C-2' secondary position, and the release of unsaturated myristic acid (Δm = 208 u) as a ketene from C-2' primary position; and b) at MS^5 stage of the selected ion at *m/z* 690 for PHAD-504, demonstrating the release of lauric acid (Δm = 182 u) as a ketene from the C-2' secondary position. 6

Figure S6. ESI-ion trap mass spectra obtained at MS^3 and MS^4 stages of selected precursor ions at a) *m/z* 1500 and b) *m/z* 1005 for 3D(6-acyl)-PHAD, demonstrating the structural relationship between $^{0,2}A_2$ and $^{0,4}A_2$ cross-ring fragment ions in 3-deacyl lipid A.......... 7

Figure S7. ESI-ion trap mass spectra obtained at MS^3 and MS^4 stages of selected precursor ions at a) *m/z* 1516 and b) *m/z* 1231 for PHAD, demonstrating the structural relationship between $^{0,2}A_2$ and $^{0,4}A_2$ cross-ring fragment ions in 3-acyl lipid A.......... 8

Scheme S1. Alternative fragmentation pathways for the elimination of the C-2 branched acyl chain as an acid in lipid A. 9

Scheme S2. Alternative fragmentation pathways for the elimination of the C-2' branched acyl chain as an acid in lipid A.......... 10

Scheme S3. Alternative fragmentation pathway for the C-2' branched acyl chain as a ketene in lipid A. 11

Scheme S4. Proposed mechanism leading to the release of the C-2' primary fatty acid as ketene in lipid A.......... 12

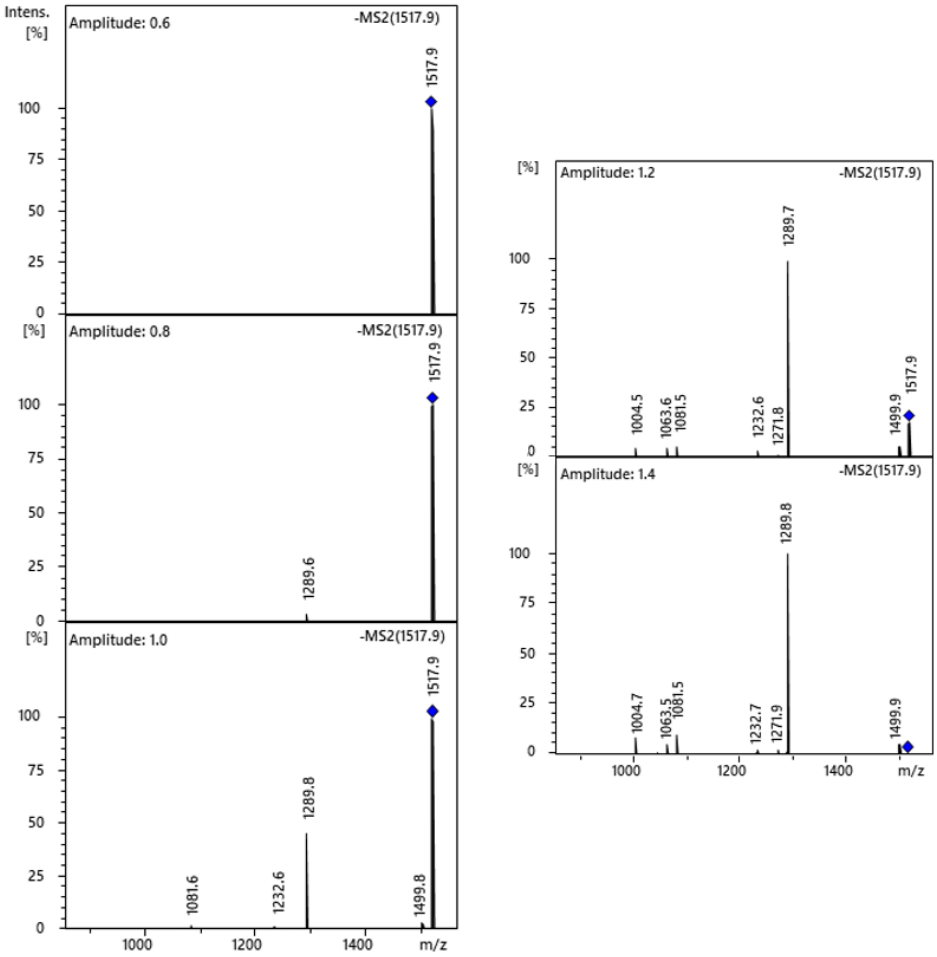

Figure S1. ESI-ion trap MS^2 mass spectra of the deprotonated form of the 3D-PHAD lipid A molecule (precursor ion *m/z* 1518) applying different RF amplitudes.

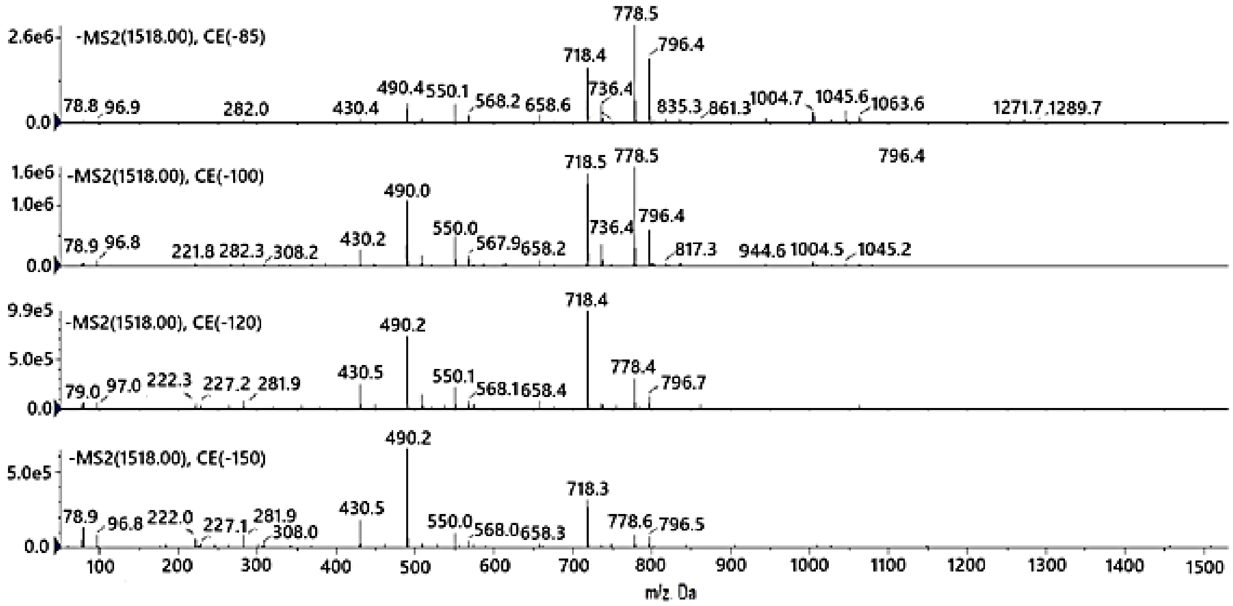

Figure S2. ESI-QqQ MS^2 mass spectra of the deprotonated form of the 3D-PHAD lipid A molecule (precursor ion *m/z* 1518) applying different collision energies (CE).

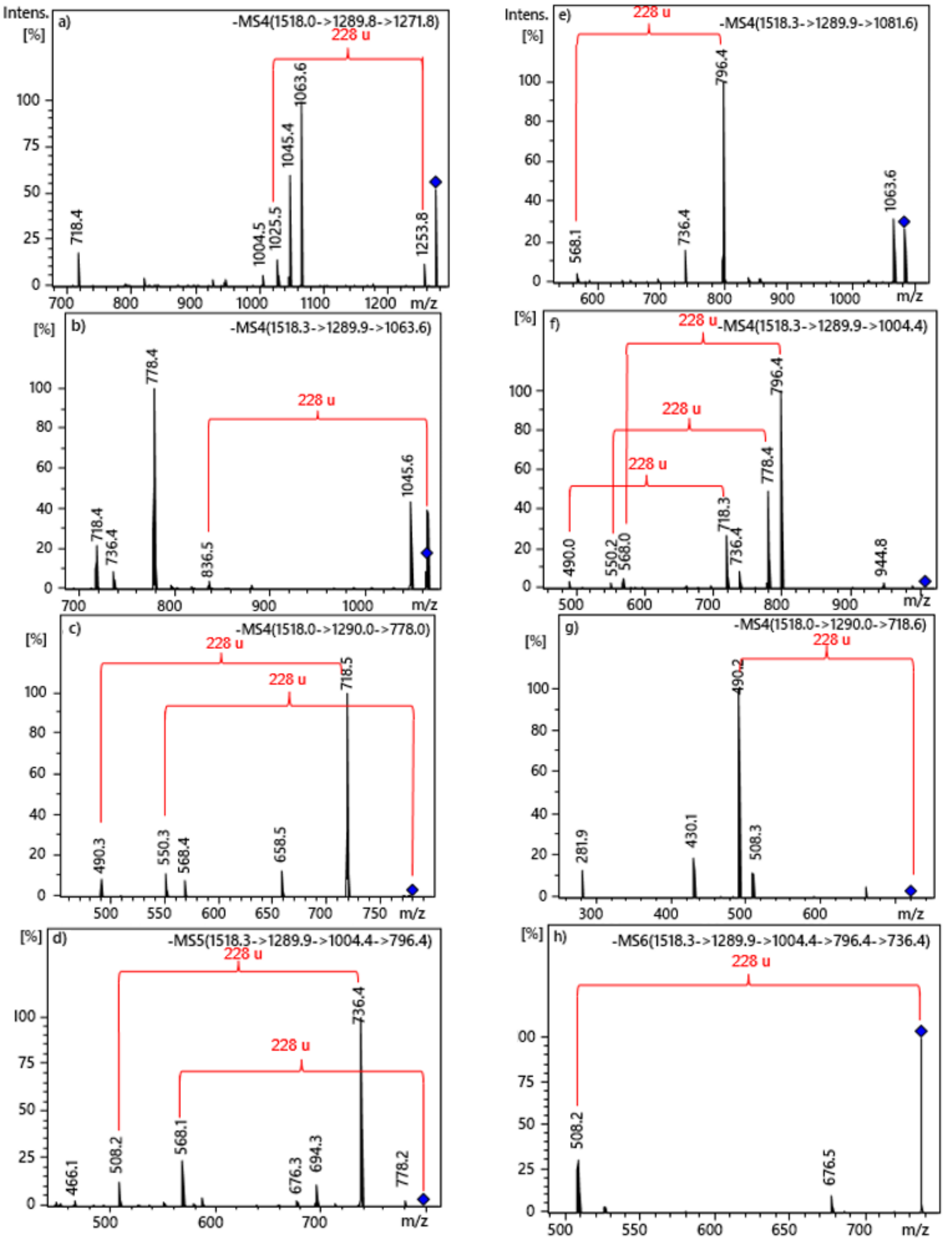

Figure S3. ESI-ion trap mass spectra obtained at MS^4, MS^5 and MS^6 stages for several fragment ions of 3D-PHAD, demonstrating the release of myristic acid (Δm = 228u) as an acid from the C-2’ secondary position.

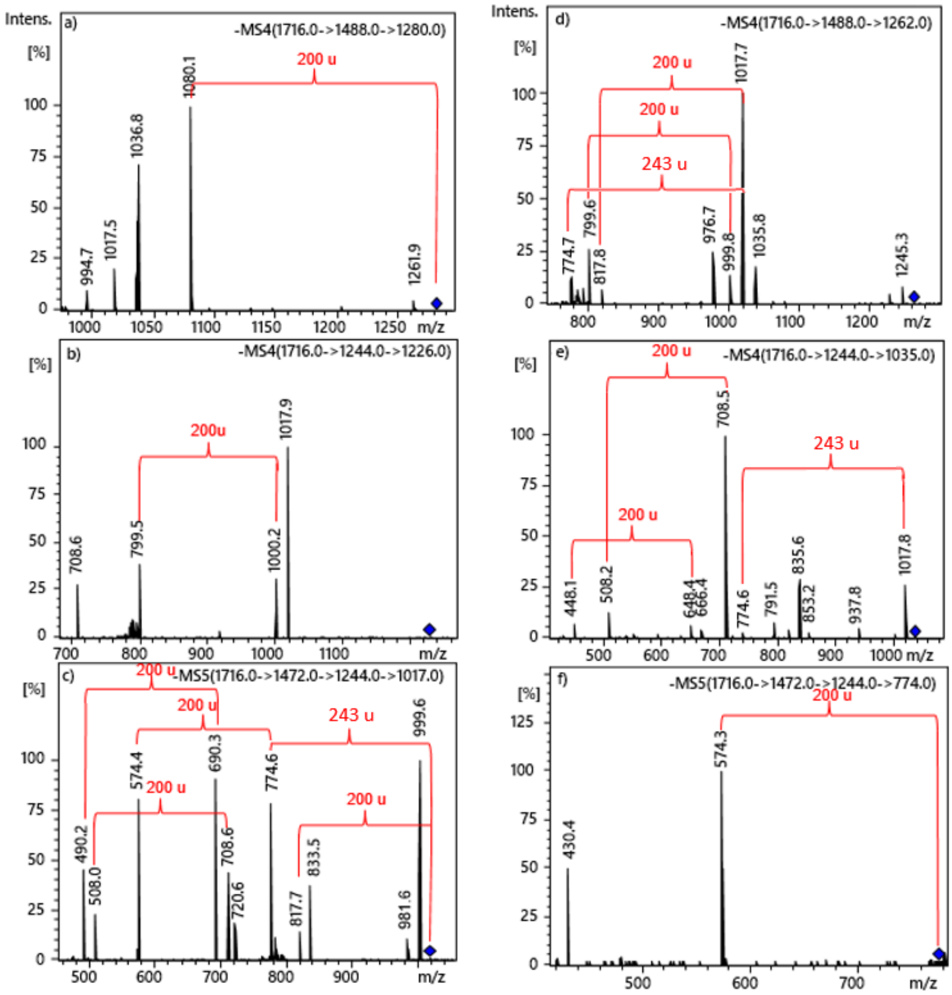

Figure S4. ESI-ion trap mass spectra obtained at MS^4 and MS^5 stages for several fragment ions of PHAD-504, demonstrating the release of lauric acid (Δm = 200 u) as an acid from the C-2' secondary position, and the release of hydroxymyristic acid amide from the C-2 primary position (Δm = 243 u).

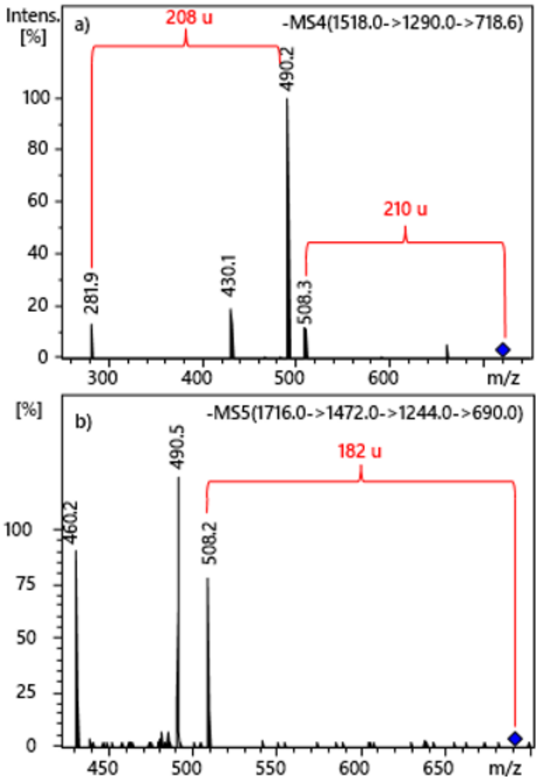

Figure S5. ESI-ion trap mass spectra obtained at a) MS^4 stage of the selected ion at *m/z* 718 for 3D-PHAD, demonstrating the release of myristic acid (Δm = 210 u) as a ketene from the C-2' secondary position, and the release of unsaturated myristic acid (Δm = 208 u) as a ketene from C-2' primary position; and b) at MS^5 stage of the selected ion at *m/z* 690 for PHAD-504, demonstrating the release of lauric acid (Δm = 182 u) as a ketene from the C-2' secondary position.

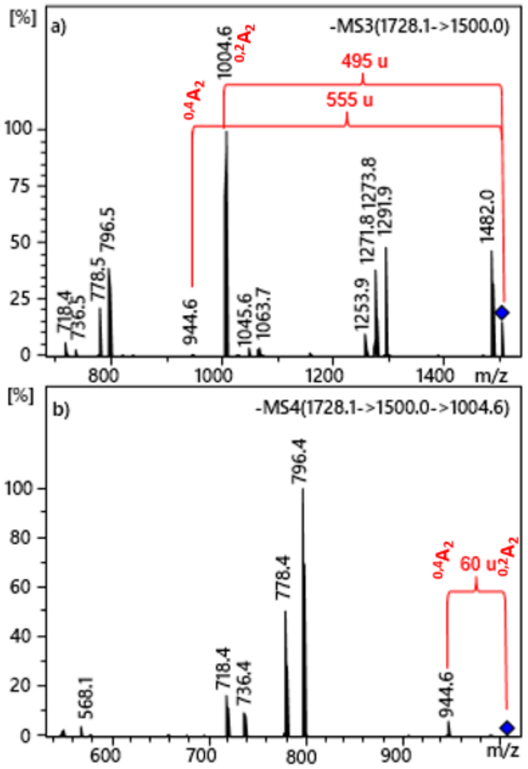

Figure S6. ESI-ion trap mass spectra obtained at MS^3 and MS^4 stages of selected precursor ions at a) *m/z* 1500 and b) *m/z* 1005 for 3D(6-acyl)-PHAD, demonstrating the structural relationship between $^{0,2}A_2$ and $^{0,4}A_2$ cross-ring fragment ions in 3-deacyl lipid A.

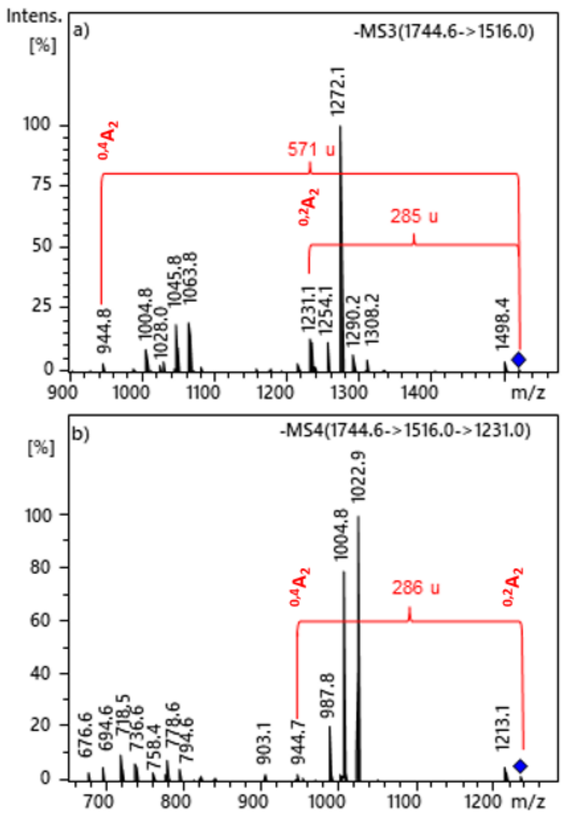

Figure S7. ESI-ion trap mass spectra obtained at MS^3 and MS^4 stages of selected precursor ions at a) *m/z* 1516 and b) *m/z* 1231 for PHAD, demonstrating the structural relationship between $^{0,2}A_2$ and $^{0,4}A_2$ cross-ring fragment ions in 3-acyl lipid A.

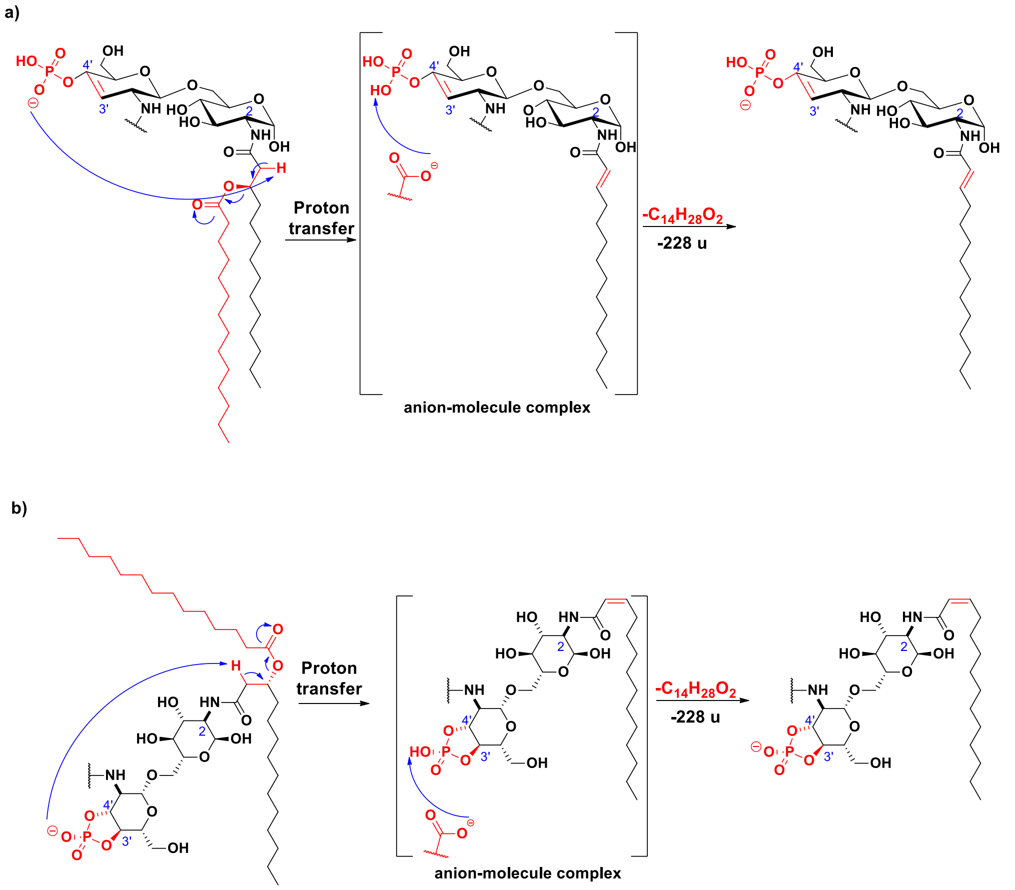

Scheme S1. Alternative fragmentation pathways for the elimination of the C-2 branched acyl chain as an acid in lipid A.

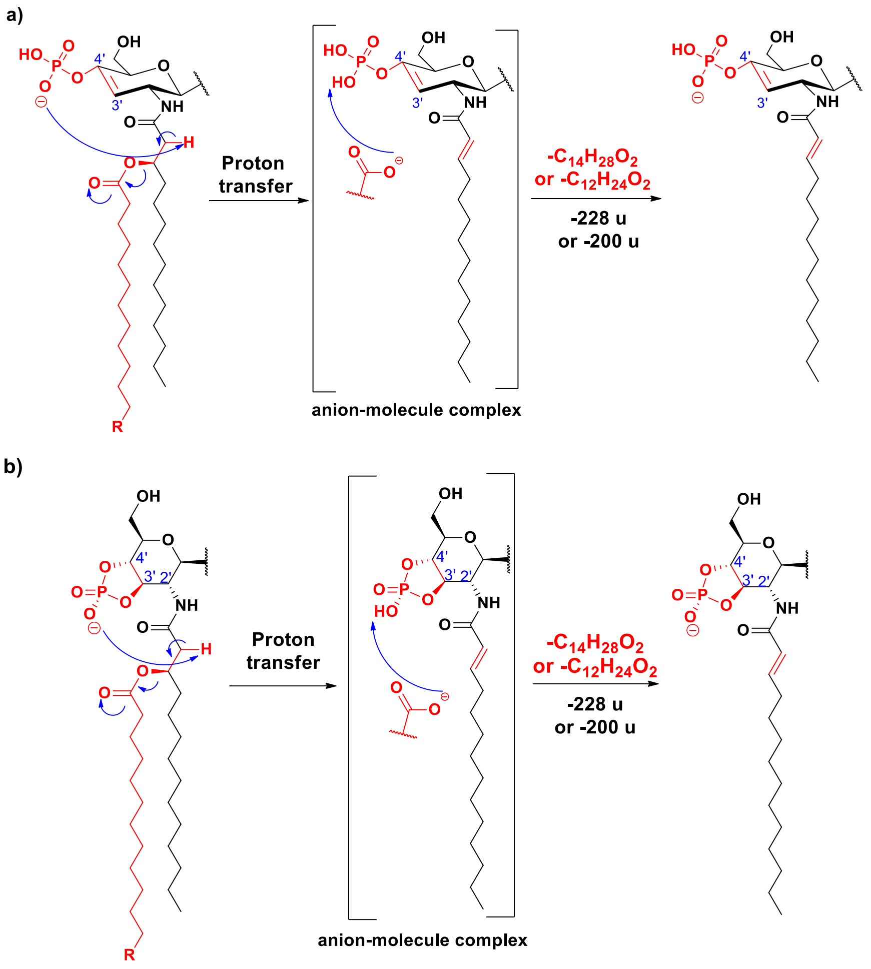

Scheme S2. Alternative fragmentation pathways for the elimination of the C-2’ branched acyl chain as an acid in lipid A.

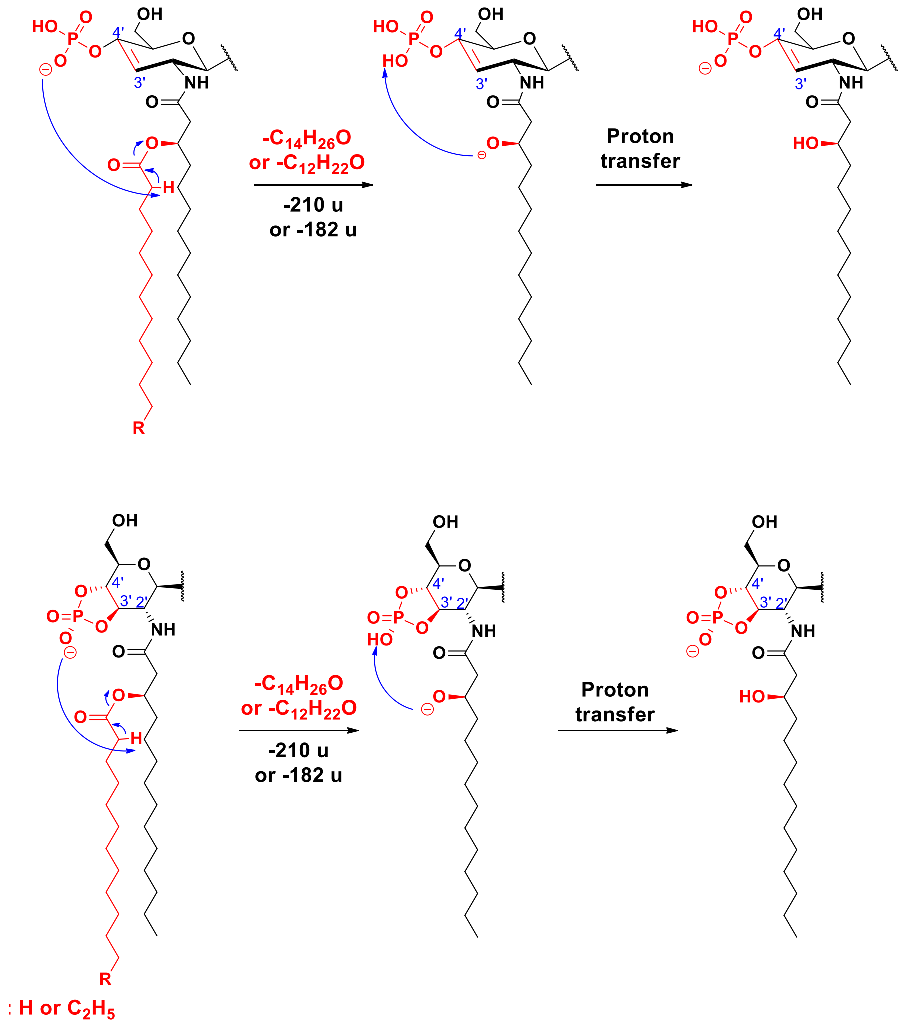

Scheme S3. Alternative fragmentation pathway for the C-2’ branched acyl chain as a ketene in lipid A.

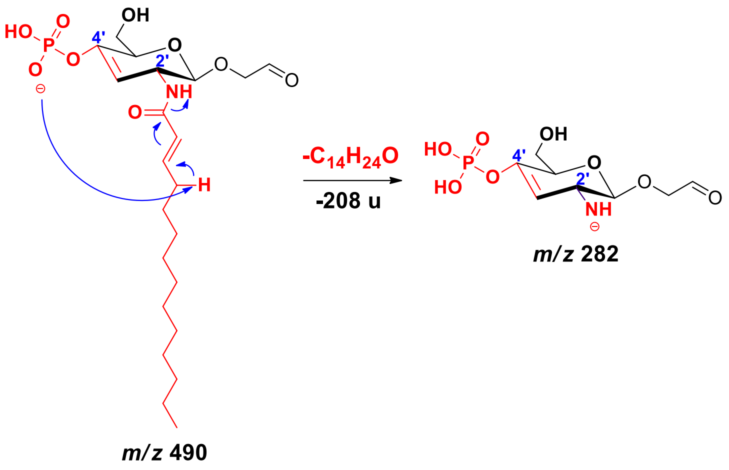

Scheme S4. Proposed mechanism leading to the release of the C-2’ primary fatty acid as ketene in lipid A.
